# Phyto-Phospholipid Conjugated Scorpion Venom Nanovesicles as Promising Carrier That Improves Efficacy of Thymoquinone against Adenocarcinoma Human Alveolar Basal Epithelial Cells

**DOI:** 10.3390/pharmaceutics13122144

**Published:** 2021-12-13

**Authors:** Hani Z. Asfour, Usama A. Fahmy, Waleed S. Alharbi, Alshaimaa M. Almehmady, Abdulmohsin J. Alamoudi, Singkome Tima, Rasha A. Mansouri, Ulfat M. Omar, Osama A. A. Ahmed, Shadi A. Zakai, Ahmed A. Aldarmahi, Alaa Bagalagel, Reem Diri, Nabil A. Alhakamy

**Affiliations:** 1 Department of Medical Microbiology and Parasitology, Faculty of Medicine, King Abdulaziz University, Jeddah 21589, Saudi Arabia; hasfour@kau.edu.sa (H.Z.A.); szakai@kau.edu.sa (S.A.Z.); 2 Department of Pharmaceutics, Faculty of Pharmacy, King Abdulaziz University, Jeddah 21589, Saudi Arabia; wsmalharbi@kau.edu.sa (W.S.A.); amnalmehmady@kau.edu.sa (A.M.A.); oaahmed@kau.edu.sa (O.A.A.A.); nalhakamy@kau.edu.sa (N.A.A.); 3 Center of Research Excellence for Drug Research and Pharmaceutical Industries, King Abdulaziz University, Jeddah 21589, Saudi Arabia; 4 Department of Pharmacology, Faculty of Pharmacy, King Abdulaziz University, Jeddah 21589, Saudi Arabia; ajmalamoudi@kau.edu.sa; 5 Department of Medical Technology, Faculty of Associated Medical Sciences, Chiang Mai University, Chiang Mai 50200, Thailand; singkome@gmail.com; 6 Department of Biochemistry, Faculty of Sciences, King Abdulaziz University, Jeddah 21589, Saudi Arabia; amansouri@kau.edu.sa (R.A.M.); uomer@kau.edu.sa (U.M.O.); 7 College of Sciences and Health Professions, King Saud bin Abdulaziz University for Health Sciences, Jeddah 21423, Saudi Arabia; aldarmahia@ksau-hs.edu.sa; 8 Department of Pharmacy Practice, Faculty of Pharmacy, King Abdulaziz University, Jeddah 21589, Saudi Arabia; abagalagel@kau.edu.sa (A.B.); rdiri@kau.edu.sa (R.D.); 9 Mohamed Saeed Tamer Chair for Pharmaceutical Industries, Faculty of Pharmacy, King Abdulaziz University, Jeddah 21589, Saudi Arabia

**Keywords:** nanoparticles, cancer, nanovesicles, cytotoxicity

## Abstract

Lung cancer is a dangerous type of cancer in men and the third leading cause of cancer-related death in women, behind breast and colorectal cancers. Thymoquinone (THQ), a main compound in black seed essential oils, has a variety of beneficial effects, including antiproliferative, anti-inflammatory, and antioxidant properties. On the other hand, scorpion venom peptides (SV) induce apoptosis in the cancer cells, making it a promising anticancer agent. THQ, SV, and Phospholipon^®^ 90H (PL) were incorporated in a nano-based delivery platform to assess THQ’s cellular uptake and antiproliferative efficacy against a lung cancer cell line derived from human alveolar epithelial cells (A549). Several nanovesicles were prepared and optimized using factorial experimental design. The optimized phytosome formulation contained 79.0 mg of PL and 170.0 mg of SV, with vesicle size and zeta potential of 209.9 nm and 21.1 mV, respectively. The IC50 values revealed that A549 cells were significantly more sensitive to the THQ formula than the plain formula and THQ. Cell cycle analysis revealed that THQ formula treatment resulted in significant cell cycle arrest at the S phase, increasing cell population in this phase by 22.1%. Furthermore, the THQ formula greatly increased cell apoptosis (25.17%) when compared to the untreated control (1.76%), plain formula (11.96%), or THQ alone (13.18%). The results also indicated that treatment with THQ formula significantly increased caspase-3, Bax, Bcl-2, and p53 mRNA expression compared to plain formula and THQ. In terms of the inflammatory markers, THQ formula significantly reduced the activity of TNF-α and NF-κB in comparison with the plain formula and THQ only. Overall, the findings from the study proved that a phytosome formulation of THQ could be a promising therapeutic approach for the treatment of lung adenocarcinoma.

## 1. Introduction

Lung cancer is one of the most serious types of cancer. It is regarded as the greatest cause of cancer-related morbidity and mortality in men, and the third biggest cause of cancer-related death in women, trailing only breast and colorectal cancer in this category [1]. Small-cell carcinoma and non-small-cell carcinoma are the two subtypes. In the United States, lung adenocarcinoma is the most common type of lung cancer. It is an example of a non-small-cell lung cancer (NSCLC) with a significant relation to smoking, which continues to be the leading cause of lung cancer. [2,3]. There are other risk factors that lead to lung cancer, such as diet, alcohol, and air pollution [4]. There are various types of lung cancer based on the histology, such as squamous cell carcinoma and bronchioalveolar cell carcinoma [4]. Patients with lung cancer may receive different therapeutic options according to the stage of the disease, including surgery, radiation, chemotherapy, targeted therapy, and immunotherapy. However, current therapeutic interventions have some limitations, such as low safety and tolerability profile. For example, prolonged use of targeted treatments leads to the development of acquired resistance, and then reducing their efficacy [5].

Black seeds, known scientifically as *Nigella sativa* from the family *Ranunculaceae*, are abundant in beneficial volatile oils. The United States Food and Drug Administration classifies black seed oil as “Generally Recognized as Safe” [6,7,8]. Thymoquinone (THQ) is a monoterpene known as 2-isopropyl-5-methylbenzo-1, 4-quinone, and is one of the major compounds in the essential oils of Nigella sativa, with other compounds, e.g., p-cymene, carvacrol, and thymol. THQ has a wide range of beneficial effects, such as antiproliferative, anti-inflammatory, antioxidant, antimicrobial, anticoagulant, immunomodulatory, and hepatoprotective effects [6,7,8]. In terms of cancer treatment, numerous studies have been undertaken to elucidate the underlying mechanism of action of THQ as an anticancer or chemopreventive agent in a variety of cancer cell lines and animal models. These studies revealed that THQ has a variety of important mechanisms of action in cancer treatment, such as inducing apoptosis in cancer cells by generating reactive oxygen species (ROS), interfering with DNA structure and synthesis, acting as an immunomodulator, and targeting carcinogenic signaling pathways [9,10,11]. Nanotechnology-based delivery platforms have demonstrated promise in the treatment of cancer due to their enhanced permeability and retention (EPR) properties, which facilitate drug targeting to cancerous cells [12].

Nanovesicles are highly promising systems for the delivery and/or targeting of drugs that can help overcome poor absorption and bioavailability of phytochemicals by boosting the penetration through biological barriers [13]. Examples of this include liposome [13], phytosome [14], and nanostructured lipid carrier (NLC) [15], etc. Nanocarriers have a crucial role in preserving bioactive phytochemicals from oxidation and degradation compared to typical phytochemical delivery, and therefore maintaining and increasing their long-term benefits and stability [16,17]. The large surface area of nanoparticles aids in the effective delivery of active substances [18]. The phyto-phospholipid nanovesicle (i.e., phytosome) is composed of plant extract incorporated into phospholipids. In nonpolar solvents, the bioactive herbal extracts’ moiety interacts via H-bonding with the phosphate group of the phospholipid matrix to form lipid vesicles [19]. The phospholipid moiety of the phytosomal nanovesicle is similar in structure to the phospholipids that form the cell membrane (i.e., biological barrier), which can enhance passage through the cell membrane and increase cellular uptake [20]. The incorporation of poorly soluble phytochemicals into the phytosomal delivery system has an important effect on the improvement of their absorption, leading to enhanced penetration and absorption across the biological membrane and better bioavailability [13,21].

Scorpion venom (SV) can lead to life-threatening medical issues and even death if injected into the human body [22]. Public health data state that around 1.5 million scorpion envenoming incidents result in 2000–3000 fatalities annually [22,23]. In terms of poisonous scorpions that are dangerous to humans, there are few families that are considered as harmful, such as the Buthidae family, Scorpionidae family, and Hemiscorpiidae family [24,25,26]. Researchers have made significant progress in the creation of more efficient therapies for scorpion envenoming, as well as the identification of a number of SV peptides with promising therapeutic benefits. Hence, SV may be a rich source of bioactive chemicals that might be used to generate new treatment options for various medical conditions [27]. SV contains a mixture of compounds that are extremely diverse and heterogeneous. The most investigated compounds are small SV peptides that gain importance due to their bioactivity. There are various therapeutic prospects for SV-derived peptides, such as antimicrobial activity, anticancer activity, and anti-inflammatory activity [28,29,30]. Additionally, SV has the potential to be a good cancer treatment agent due to the damage it exerts on cancer cells by interfering with the cell cycle, serving as a proliferative inhibitor. By increasing nitric oxide production and caspase-3, as well as depolarizing the mitochondrial membrane, it induces apoptosis and aggravates cancer cells [31,32].

In this work, we investigated the effect of using nanovesicles (i.e., phytosomes) as a delivery platform for THQ and SV in enhancing the cellular uptake and efficacy of THQ as an antiproliferative against a lung cancer cell line model derived from human alveolar epithelial cells (A549).

## 2. Materials and Methods

### 2.1. Materials

THQ and SV were obtained from Sigma-Aldrich Inc. (St. Louis, MO, USA). Phospholipon^®^ 90H (hydrogenated phosphatidylcholine from soybean origin, content 90%) was obtained as a gift sample from Lipoid GmbH (Ludwigshafen, Germany). The human tumor cell line adenocarcinomic human alveolar basal epithelial cells (A549) used in this study were obtained from the VACSERA (Giza, Egypt) cell culture unit, which were originally acquired from ATCC (Manassas, VA, USA).

### 2.2. Experimental Design and Optimization of THQ–PL–SV Phytosomes

3^2^ Factorial experimental design was implemented to formulate and optimize THQ–PL–SV nanovesicles. PL concentration (mg, X_1_) and SV concentration (mg, X_2_) were considered as independent variables, while vesicle size (nm, Y_1_) and zeta potential (mV, Y_2_) were investigated as response parameters. The coded levels of each factor, nominated as (−1, 0, +1), and their actual values are listed in Table 1. The design yielded 18 formulations, where the combinations of variables’ levels in each formulation are compiled in Table 2. Statgraphics software (Statgraphics Technologies, Inc., Warrenton, VA, USA) was used to statistically analyze the responses data. Model fit statistics were applied for the selection of the best fitting sequential model (amongst linear, two-factor interaction, or quadratic) for each response based on the predicted and adjusted determination coefficients (R^2^). The equations representing the selected optimal model for both responses were generated. Analysis of variance (ANOVA) was applied to estimate the significance of the studied variables on the measured responses and the possible interaction between them at *p* < 0.05. Three-dimensional and interaction plots were produced to explore the interaction between the investigated variables.

### 2.3. Preparation of THQ–PL–SV Nanovesicles

THQ–PL–SV nanovesicles were prepared using reflux, followed by antisolvent precipitation, as described before with some modification [33]. Briefly, weighed amounts of THQ (25 mg) and Phospholipon^®^ 90H in the specified weight (according to the design) were dissolved in dichloromethane (20 mL). The solution was refluxed at 60 °C, and then evaporated to obtain a concentrate of about 5 mL. The concentrate was lyophilized for 72 h to obtain the THQ–PL. SV was dissolved in distilled water, utilizing the SV amounts specified in the design. The SV aqueous solution was used as the hydration medium for the dried THQ–PL to prepare the THQ–PL–SV nanovesicles that were then stored in airtight amber-colored glass containers at 4 °C until further use [34].

#### 2.3.1. Vesicle Size and Zeta Potential Determination

THQ–PL–SV size and zeta potential were determined by appropriate dilution in double-distilled water using a Zetasizer Nano ZSP particle size analyzer instrument (Malvern, UK). The results were expressed as the average of five determinations. The parameters were as follows: laser wavelength of 633 nm, scattering angle of 173, temperature of 25 °C, medium viscosity of 0.8872 cP, and medium refractive index of 1.33.

#### 2.3.2. Optimization of THQ–PL–SV Nanovesicles

To predict the optimized levels of the investigated factors, the desirability function that amalgamates both responses was calculated. The desired goals were minimizing the vesicle size and maximizing the absolute zeta potential (Table 1). Nonetheless, the predicted optimized THQ–PL–SV nanovesicles were prepared for further characterization.

### 2.4. Fourier-Transform Infrared Characterization of the Optimized THQ–PL–SV Nanovesicles

The optimized THQ–PL–SV nanovesicles and their formula components, THQ, PL, and SV spectra, were measured in the range of 4000–400 cm^−1^ using a Fourier-transform infrared (FTIR) spectrometer (Tensor 37, Bruker, Fremont, CA, USA).

### 2.5. Cytotoxicity of Optimized THQ–PL–SV

The cytotoxicity efficacy of optimized THQ–PL–SV (THQ Formula) was performed on the A549 cell line using MTT assay. For this experiment, selected cells were grown in 96-well plates at the density of 5 × 10^3^ cells per well and incubated overnight. After stabilization, cells were treated with Plain PL-SV (Plain Formula), THQ raw (THQ), and THQ–PL–SV (THQ Formula) and incubated for 24 h. Then, previously treated cells were further treated with 5.0 mg/mL MTT solution (10 µL), then incubated for 4 h at 37 °C. Additionally, the collected supernatant was dispersed in 100 mL of DMSO to solubilize the formazan crystal. Samples were analyzed employing a microplate reader at 570 nm. Studies were carried out in triplicate [35].

#### 2.5.1. Cell Cycle Analysis

To analyze the effects of samples on the cell cycle, the flow cytometry method was utilized. The cells were treated with various sample formulations: PL–SV (Plain Formula), THQ raw (THQ), and THQ–PL–SV (THQ Formula), and incubated for 24 h. After completion of incubation, cells were separated by centrifugation and fixed with 70% cold ethanol. Prior to washing of samples with PBS, samples were again separated by centrifugation. Separated cells were further stained with propidium iodide and RNAse before starting flow cytometry analysis [36,37,38].

#### 2.5.2. Analysis of Apoptosis by Annexin V Staining

In order to analyze the comparative apoptotic activity of PL–SV (Plain Formula), THQ raw (THQ), and THQ–PL–SV (THQ Formula), the Annexin V method was implemented. For this purpose, selected cells were grown in 6-well plates at the density of 1 × 10^5^ cells per well, then incubated overnight with IC50 concentration of samples for 24 h at 37 °C. All samples were then centrifuged at 200× *g* for 5 min, and collected cells were resuspended in PBS at room temperature after dual washing. Further, 10 µL Annexin V and 5 µL propidium iodide solution supernatant was dispersed in the previously prepared samples and incubated at 25 °C for 5 min. Final samples were analyzed using a flow cytometer (FACS Calibur, BD Bioscience, California, CA, USA) in triplicate [33,39].

#### 2.5.3. Real-Time Polymerase Chain Reaction (RT-PCR) for Estimation of Bcl-2, Bax, P53, Caspase 3, and TNF-α

The expression of Bcl-2, Bax, p53, caspase 3, and TNF-α was determined by using RT-PCR. The PANC1 cells were treated with TQ alone, plain formula alone, and TQ formula and incubated for the specified period of time. The cell fraction was used for the extraction of RNA and proceeded for the synthesis of cDNA. Primer for the Bcl-2, Bax, *p53*, caspase 3, and TNF-α was designed by using Gene Runner software. The prepared samples were estimated for the expression in triplicate, and the samples were normalized with the help of β actin [37,40].

#### 2.5.4. Determination of Mitochondrial Membrane Potential (MMP)

ABCAM assay kit (Abcam, Cambridge, UK) was used for the estimation of MMP. In a 96-well plate with A549 cell density, 5 × 10^3^ was incubated for 24 h in TQ alone, plain formula alone, as well as TQ formula being added into the well separately. The resultant cell mixture was kept in the dark, probe solution (tetramethylrhodamine, methyl ester) was replaced, and MMP was observed using FACS Caliber, BD Bioscience flow cytometer [41,42].

### 2.6. Statistical Analysis

The value was expressed as mean ± standard deviation (SD). One-way ANOVA, followed by Tukey’s multiple comparison test, was used for statistical analysis, where *p*-value < 0.05 was considered as significant.

## 3. Results

### 3.1. Experimental Design

In this study, the measured vesicle size and zeta potential data were fitted to the appropriate model. For each response, the predicted R^2^ of the fitting model coincides reasonably with the corresponding adjusted R^2^, see Table 3. Moreover, the R^2^ for both variables indicates that the model, as fitted, explains 99.3799% and 66.8896% of the variability in size and zeta potential, respectively [43,44].

#### 3.1.1. Effect of Variables on Vesicle Size (Y_1_)

**Figure 1:**
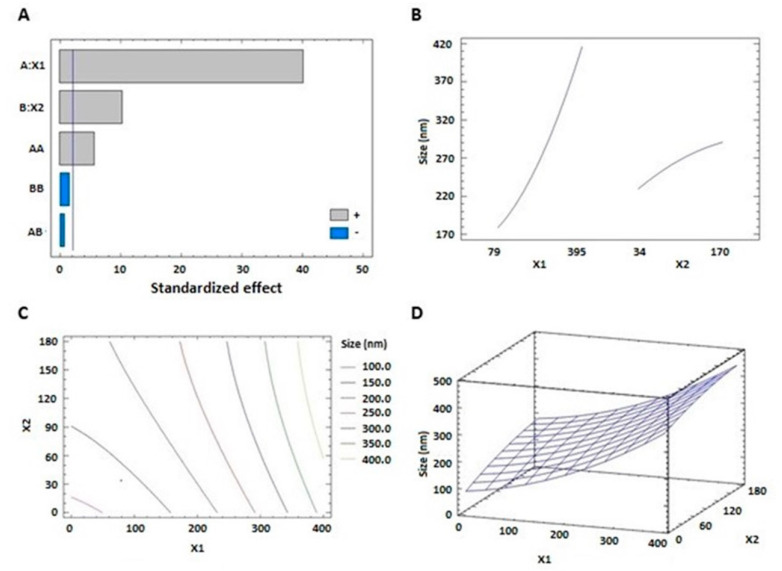
Standardized pareto chart (**A**), main effects plot (**B**), contour 2D plot (**C**), and response 3D plot (**D**) showing the effects and interaction between PL and SV amounts on the vesicle size of THQ–PL–SV phytosomes. Abbreviations: THQ, thymoquinone; PL, Phospholipon^®^ 90H; SV, scorpion venom peptide.

The prepared THQ–PL–SV phytosomes showed acceptable vesicle sizes, ranging from 143.7 to 435.6 nm (Table 2). The vesicle size of the proposed THQ–PL–SV phytosomal formulation was optimized to minimized value. The model relating the vesicle size to the studied independent variables indicated the significance of the main effects and the interactions between variables, see Table 3. The equation representing the model for the vesicle size in terms of coded factors was computed (Equation (1)). Analysis of variance (ANOVA) revealed that both PL (X_1_) and SV (X_2_) amounts exhibited a significant effect on vesicle size (*p* < 0.0001), see Table 4. In addition, the interaction term (X_1_X_1_) was statistically significant on *p* value 0.0001; detailed analysis is provided in Table 4. The standardized pareto chart (Figure 1A), main effects (Figure 1B), 2D contour (Figure 1C), and response 3D-surface (Figure 1D) plots show the influence of PL and SV amounts on the vesicle size. It was evident that increasing both factors led to an increase in the vesicle size. This observation is supported by the positive sign of both terms X1 and X2 in the previously mentioned equation. The results revealed the increase in THQ–PL–SV phytosome vesicle size with increasing both PL and SV amounts.

THQ-PL-SV phytosomes vesicle size = 86.4819 + 0.216772 × X1 + 0.853382 × X2 + 0.0011824 × X1^2 − 0.000239203 × X1 × X2 − 0.00169875 × X2^2(1)

#### 3.1.2. Effect of Variables on Zeta Potential (Y2)

**Figure 2:**
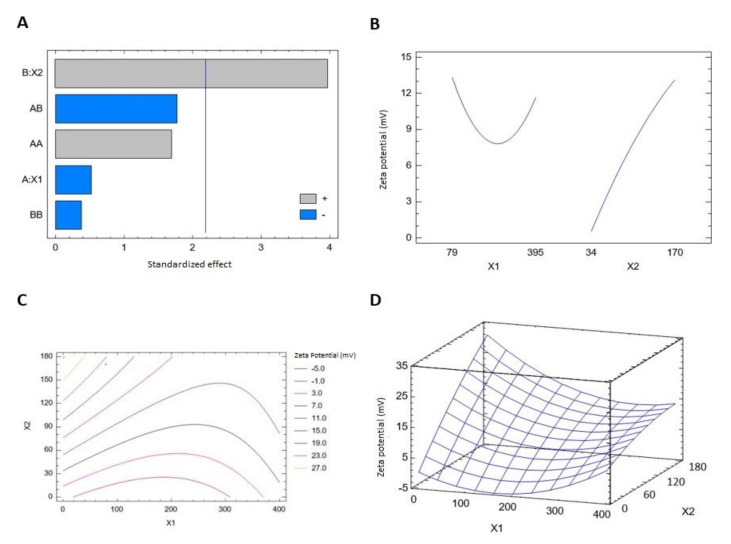
Standardized pareto chart (**A**), main effects plot (**B**), contour 2D plot (**C**) and response 3D plot (**D**) showing the effects and interaction between PL and SV amounts on the zeta potential of THQ–PL–SV phytosomes. Abbreviations: THQ, thymoquinone; PL, Phospholipon^®^ 90H; SV, scorpion venom peptide.

Zeta potential results showed that the prepared THQ–PL–SV phytosomes revealed positive surface charge ranging from 2.9 ± 0.1 to 26.9 ± 1.2 mV. The equation representing the zeta potential in terms of coded factors was computed (Equation (2)). Analysis of variance (ANOVA) revealed only SV (X2) amounts exhibited a significant effect on zeta potential (*p* value 0.0022), see Table 5. The effect of SV amount was pronounced as evidenced by the high coefficient of its linear term (X2) and lowest *p*-value. Detailed analysis is provided in Table 5. The standardized pareto chart (Figure 2A), main effects (Figure 2B), 2D contour (Figure 2C), and response 3D-surface (Figure 2D) plots show the influence of PL and SV amounts on the zeta potential. The zeta potential increases at higher SV amounts. This observation is supported by the corresponding positive sign of the term X2 in the coded equation.

THQ-PL-SV phytosomes zeta potential = 0.04375 − 0.0605222 × X1 + 0.213297 × X2 + 0.000185267 × X1^2 − 0.000318783 × X1 × X2 − 0.00022167 × X2^2(2)

Analysis of variance (ANOVA), using sum of squares Type III partial, revealed that both PL (X1) and SV (X2) amounts exhibited a significant effect on zeta potential, see Table 5. The effect of SV amount was more pronounced than that of PL amount, as evidenced by the higher coefficient of its linear term (X2) and lowest *p*-value.

#### 3.1.3. Optimization of THQ–PL–SV Phytosome Formulation

The optimized levels of PL and SV amounts that could achieve the set goals of minimizing vesicle size and maximizing zeta potential were predicted by a numerical optimization technique. The multiple response optimization was executed and the optimized levels of 79.0 mg and 170.0 mg for PL and SV amounts, respectively, are supposed to achieve the goals upon combination. The measured vesicle size and zeta potential values of 209.9 nm and 21.1 mV were in good harmony with the predicted values of 203.75 nm and 20.38 mV. The relatively low percentage error of less than 5% for both responses confirm the design suitability and the validity of the optimization technique.

### 3.2. (FTIR) Characterization of the Optimized THQ–PL–SV Nanovesicles

**Figure 3:**
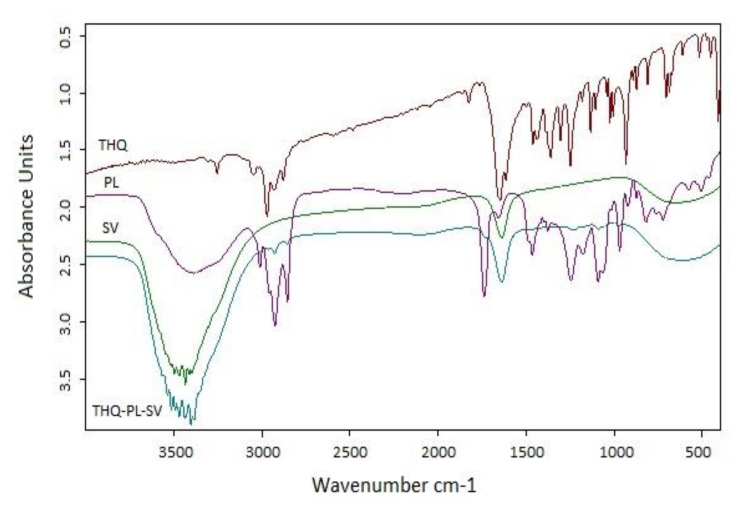
FTIR spectra of THQ, PL, SV, and optimized THQ–PL–SV. Abbreviations: THQ, thymoquinone; PL, Phospholipon^®^ 90H; SV, scorpion venom peptide.

FTIR data were utilized to investigate the interaction among components of the optimized THQ–PL–SV nanovesicles. Results (Figure 3) revealed that FTIR spectrum of THQ revealed a characteristic absorption band at 3000–2800 cm^−1^ corresponding to aliphatic C–H stretching of the isopropyl and CH3 groups. Moreover, it has an intense band at ≈ 1715 cm^−1^ for C=O stretching. PL spectrum assigned a characteristic C–H stretching signal present in the long fatty acid chain at 2925 and 2855 cm^−1^. In addition, a C=O stretching band at 1738 cm^−1^ in the fatty acid ester, and an ester C–O stretching band at 1245 cm^−1^, a P=O stretching band at 1236 cm^−1^, a P–O–C stretching band at 1091 cm^−1^, and a −N+(CH3)3 stretching at 970 cm^−1^ were also observed in the spectrum. SV spectra showed a very clear characteristic band at 3400–3300 cm^−1^, which was attributed to the amino group of amino acids, and 1620 cm^−1^ due to the amidic C=O group of the peptide backbone. In the spectrum of THQ–PL–SV, the tip of the peak around 3400 cm^-1^ has a shift to 3300 cm^−1^ and becomes wider with increased relative intensity, indicating an enhancement of hydrogen bonding interactions. Moreover, in THQ–PL–SV spectra, the peaks for N–H bending vibration of amino group at 1600 cm^−1^ and the amide carbonyl stretch at 1650 cm^−1^ diminished the C=O peaks of THQ and PL, which indicates involvement of these groups in the interaction with SV amino and amide groups. Moreover, both bands of THQ and PL before 3000 cm^−1^ were found to have almost disappeared, indicating a possibility of hydrophobic interactions of the two compounds through these aliphatic parts.

### 3.3. Determination of IC_50_ Values

**Figure 4:**
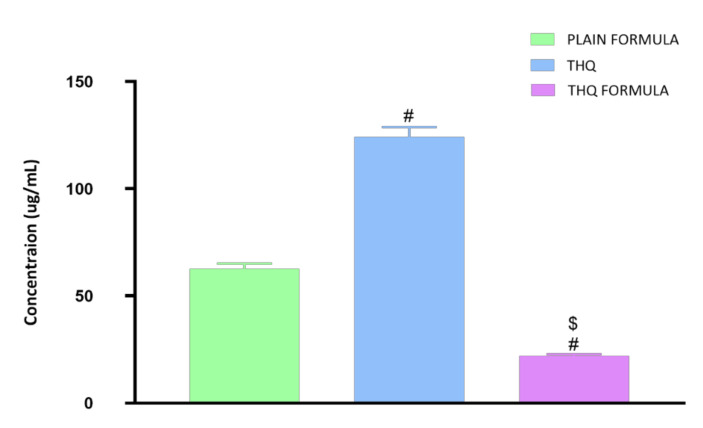
Representation of the IC50 values of plain formula, THQ, and THQ formula in A549 cells. # Significantly different from plain formula at *p* < 0.05. $ Significantly different from THQ at *p* < 0.05.

The cytotoxic activity of the THQ formula was determined by the cell-killing performance in the MTT assay on the A549 cell line. As can be seen from Figure 4, the THQ formula was highly effective in inducing cell death, with an IC50 value of 21.99 µg/mL. A549 cells were significantly more sensitive to the THQ formula compared to the plain formula and THQ, as indicated by their IC50 values of 62.74 µg/mL and 124.2 µg/mL, respectively.

### 3.4. Cell Cycle Analysis

**Figure 5:**
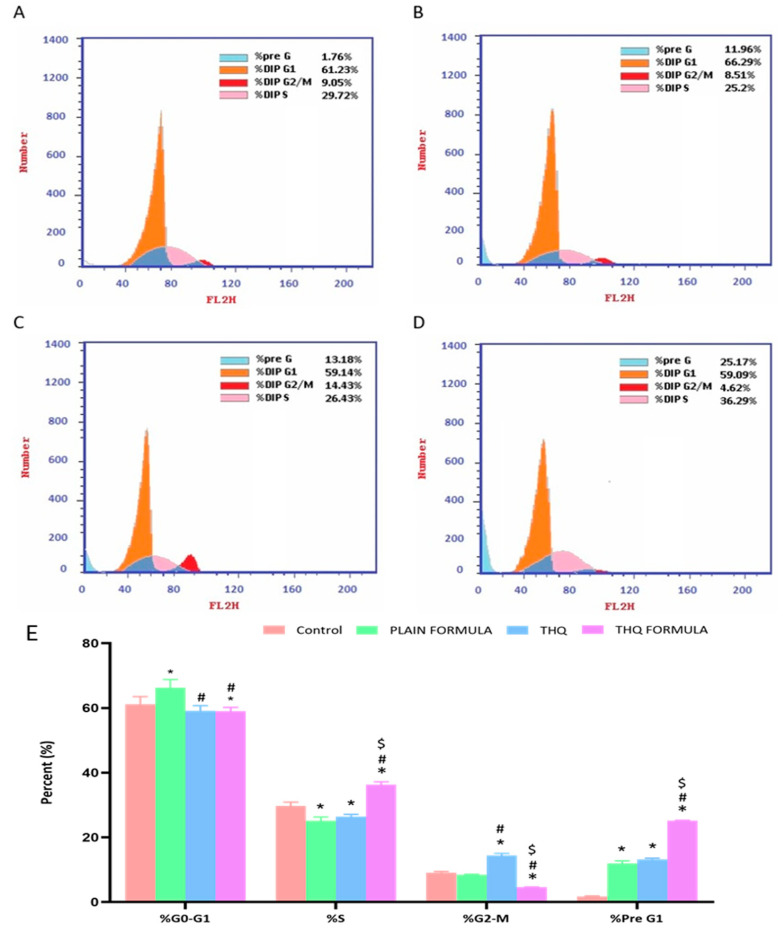
Flow cytometric analysis of control (**A**), plain formula (**B**), THQ (**C**), and THQ formula (**D**) treated cells and the percentages of cells in the G1, S, and G2/M phases of the cell cycle (**E**). * Significantly different from control at *p* < 0.05, # significantly different from plain formula at *p* < 0.05. $ Significantly different from THQ at *p* < 0.05.

Cell cycle distribution analysis was carried out to investigate the potential of the THQ formulation in hindering the cell cycle progression of A549 cells. As demonstrated in Figure 5, significant and differential alterations in cell cycle phases were detected when the cells were treated with the plain formula, THQ, and the THQ formula. In A549 cells, plain formula significantly increased cell population in G0/G1 phase by 8.2%, while THQ treatment significantly increased the cell population in G2/M phase by 59.4% (Figure 5). Treatment with THQ formula induced significant cell cycle arrest at the S phase, increasing cell population in this phase by 22.1%. The induced S cell phase arrest was accompanied by a reciprocal decrease in the cell population in the G2/M phase by 48.9% (Figure 5). Relative to the control values, exposure of A549 cells to the THQ formula resulted in significant cell death, as indicated by the 1330% increase in pre-G cell population compared to only 10.4% and 90.9% increase associated with the plain formula and THQ.

### 3.5. Apoptotic Assessment Using Annexin V

**Figure 6:**
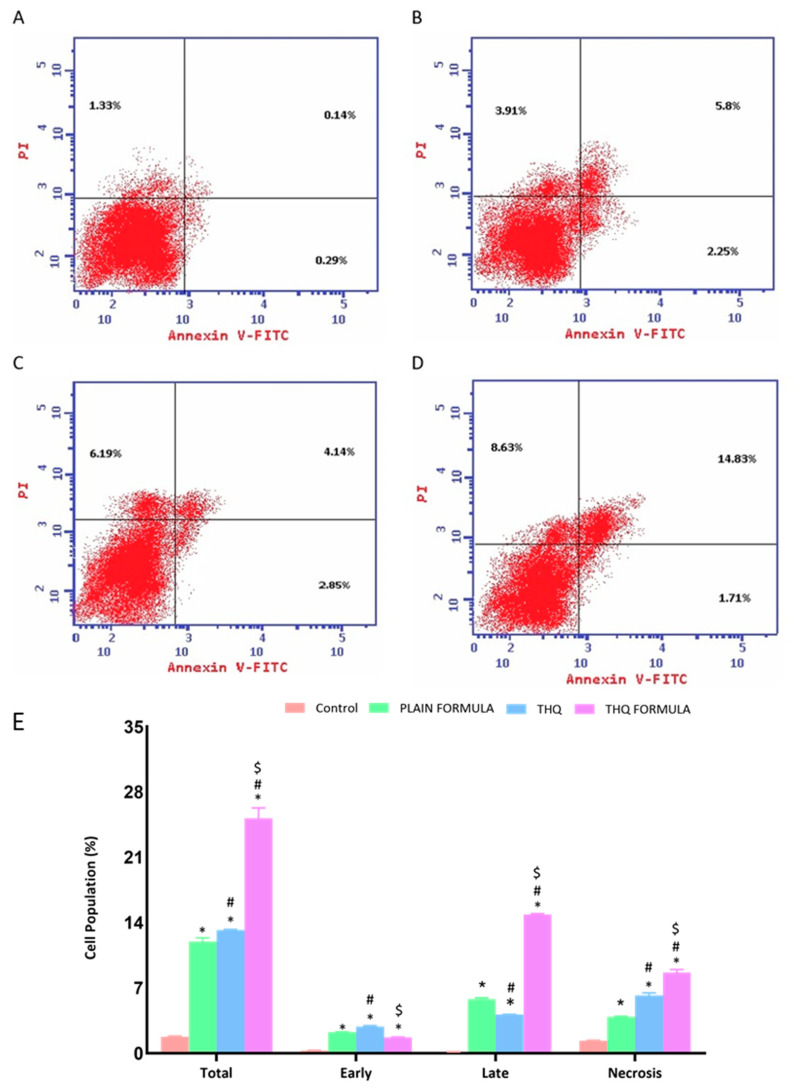
Assessment of A549 cell death in control (**A**), plain formula (**B**), THQ (**C**), and THQ formula (**D**) treated cells and the percentages of cells in early, late, and total cell death (**E**) following Annexin V staining. * Significantly different from control at *p* < 0.05. # Significantly different from plain formula at *p* < 0.05. $ Significantly different from THQ at *p* < 0.05.

The induction of apoptosis (early, late, and total) and necrosis was also assessed to examine the apoptotic potential of the THQ formula. It can be seen from Figure 6 that exposure of THQ within the novel formulation significantly induced apoptosis (25.17%), as shown in Figure 6D, when compared to the untreated control (1.76%) (Figure 6A), plain formula (11.96%) (Figure 6B), or THQ (13.18%) (Figure 6C). Additionally, significant necrotic cell death was observed following treatment with the plain formula, THQ, and the THQ formula, when compared to the necrosis in control cells, as shown in Figure 6E.

### 3.6. Assessment of mRNA Expression of Caspase-3, Bax, Bcl-2, and P53

**Figure 7:**
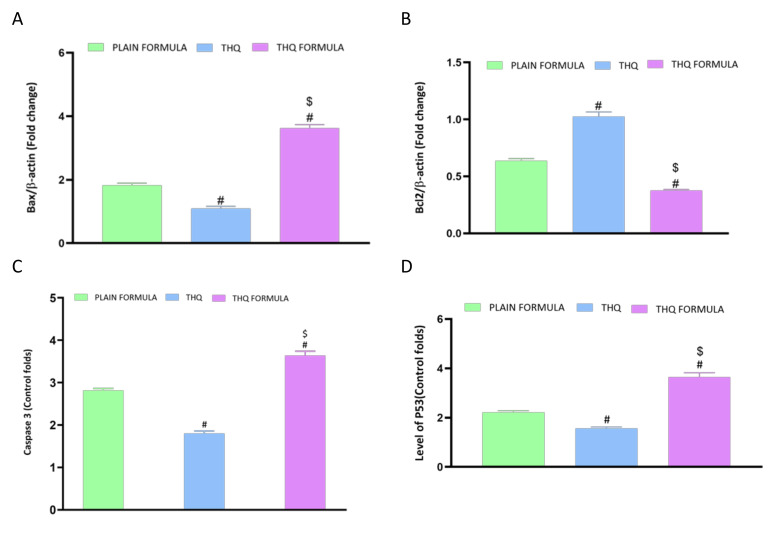
Effect of the THQ formula on mRNA expression of (**A**) Bax, (**B**) Bcl-2, (**C**) caspase-3, and (**D**) p53. Data are presented as mean ± SD (*n* = 3). #: Significantly different from plain formula at *p* < 0.05. $: Significantly different from THQ at *p* < 0.05.

The apoptotic potential of the THQ formula was also explored by assessing the mRNA expression of Bax, Bcl-2, caspase-3, and p53 in A549cells. As demonstrated in Figure 7A, the mRNA expression of Bax was significantly increased by 261.9% following treatment with the THQ formula. Treatment with this formula caused a twofold and threefold increase in Bax mRNA expression relative to the increase induced by the plain formula and THQ, respectively. In contrast to the increase observed with Bax mRNA levels, treatment with plain formula and THQ formula was associated with a 36.4% and 62.5% decrease in the mRNA expression of Bcl-2, respectively (Figure 7B). Yet, treatment with THQ did not induce a notable decrease in Bcl-2 mRNA expression. Figure 7C shows the analysis of caspase-3 levels, where significant enhancement in caspase-3 content was detected in the THQ-formula-treated A549 cells. Relative to control values, the increase in caspase-3 was about 264.1%, compared to the increase of 181.5% and 80% induced by the plain formula and THQ, respectively. The mRNA expression levels of p53 were also assessed to investigate its role in apoptosis induced by the THQ formula. As can be seen from Figure 7D, treatment of A549 cells with the THQ formula led approximately to an increase of 64.2% and 132.8% in the mRNA expression of p53 relative to the values induced by the plain formula and THQ, respectively.

### 3.7. MMP Assessment

**Figure 8:**
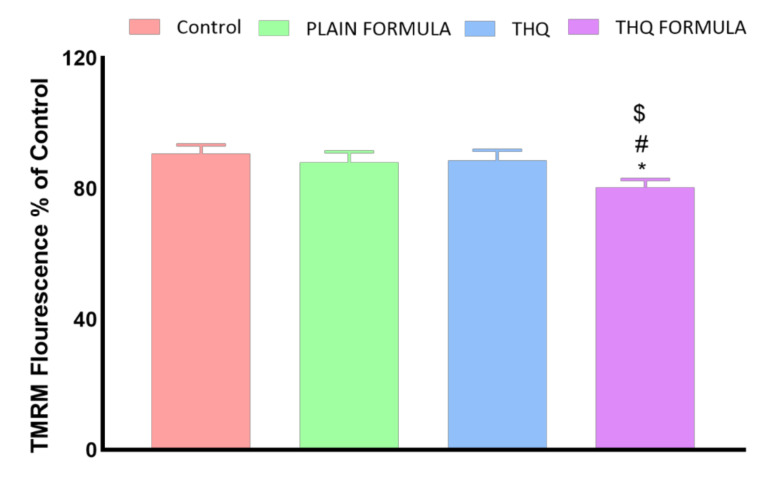
Effect of the THQ formula on mitochondrial membrane potential (MMP) in A549 cells. Data presented in bar charts are mean ± SD (*n* = 3). *, #, or $: Statistically different from control, plain formula, or THQ, respectively at *p* < 0.05.

The following set of experiments was carried out to investigate whether mitochondrial events contribute to apoptosis induced by the THQ formula. As Figure 8 shows, a significant loss of MMP was only detected in cells treated with the THQ formula. In contrast, treatment with the plain formula and THQ did not result in any significant MMP changes.

### 3.8. Assessment of Inflammatory Markers

**Figure 9:**
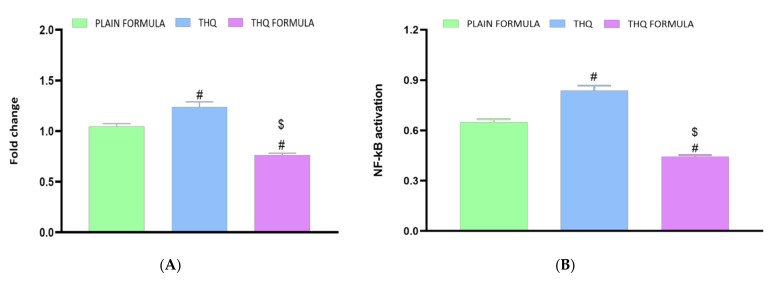
Effect of the THQ formula on the mRNA expression of TNF-α (**A**) and the activation of NF-κB (**B**) in A549 cells. Data presented in bar charts are mean ± SD (*n* = 3). # or $: Statistically different from plain formula or THQ, respectively at *p* < 0.05.

The anti-inflammatory activity of the THQ formula was also assessed based on the mRNA expression of TNF-α and the activity of NF-κB in A549 cells. Figure 9A shows that treatment with the THQ formula significantly reduced the expression of TNF-α by 23.6% relative to the control values. In contrast to the THQ formula, no significant changes were detected in cells treated with the plain formula, while THQ induced an increase in the mRNA expression of TNF-α. Similarly, NF-κB activity was decreased by 55.7% relative to the control when the cells were treated with the THQ formula (Figure 9B). Yet, treatment with the plain formula and THQ only inhibited NF-κB activation by 35.3% and 16.3%, respectively.

## 4. Discussion

Recognizing the variables that might have an impact on the nanoparticulate system characteristics is essential. Factorial design offers a privilege concerning this issue, as it possesses the ability to analyze the influence of different factors concurrently. Recently, a focus on the use of lipidic vesicular systems for cancer therapy has emerged. A particle size less than 400 nm was reported to be appropriate for preferential distribution within solid cancerous masses [45,46]. The prepared THQ–PL–SV phytosomes showed acceptable vesicle sizes ranging from 143.7 to 435.6 nm (Table 2). However, the favored buildup of nanovesicular systems within tumors and the consequent therapeutic effectiveness might be opposed by inefficacious permeation that could be attributed to pathological conditions developed by malignancy [47]. Minimizing vesicle size could result in boosting tumor penetration via extending the surface area available for permeation [48]. Therefore, the vesicle size of the proposed THQ–PL–SV phytosomal formulation was optimized to minimized value. The increase in size with increasing PL amount coincides appropriately with the previously reported pattern for vesicular systems size [49,50,51]. For example, Alhakamy et al. [33] reported higher icariin phytosome vesicular size with higher icariin to PL molar ratio. In addition, the increase in size at higher SV amounts could be explained on the basis of the positive charge of the SV peptides. Accordingly, at higher SV amounts, there is an increased induced repulsion between phospholipid vesicular bilayer by the increased positive charge. This increase in spacing is caused by propelling the phospholipid polar heads, resulting in size increase. Previous studies have also reported higher size of cationic vesicular systems relative to neutral ones [52,53]. Zeta potential gives a quantitative estimate for the surface charge of the nanoparticulate systems. Cationic nanovesicles are reported to have pronounced penetration and consequent retention in cancerous tissue and vasculature in comparison to neighboring tissues [54,55,56]. Therefore, scorpion venom was used as a positive charge inducer for surface functionalization of the proposed phytosomes. All the prepared THQ–PL–SV phytosomes showed positive surface charge. The pronounced effect of SV amount on the zeta potential could be explained on the basis of the cationic charge on the scorpion venom peptides and, consequently, its role in imparting a positive charge to the phytosomal surface [57,58].

THQ exerts its antiproliferative effects via induction of apoptosis and cell cycle arrest. Similar cytotoxic effects were also observed with SV; therefore, it would be a good candidate to improve the activity of THQ against lung cancer [59,60]. The novel THQ formula developed in this study showed significant cytotoxicity against the A549 lung cancer cells. This novel formula significantly decreased the IC50 of THQ by more than 82%, indicating that the PL–SV phytosomes act as an effective delivery system for THQ.

These results are consistent with the cytotoxic activity of THQ in human lung cancer cell lines [61]. The improved cytostatic activity of THQ formula could be attributed to the enhanced permeability of the novel formulation. Free THQ is passively transported across the cell membrane, whereas THQ incorporated into the novel phytosomes appears to be internalized via endocytosis. Furthermore, phytosomes are known to facilitate the delivery of amphiphilic agents across the lipid-rich bio-membrane of cells, hence increasing their intracellular concentrations [62,63].

The apoptotic activity of THQ, when incorporated with PL–SV phytosomes, was found to be significantly enhanced relative to the plain formula and free THQ. It appears that THQ phytosomes differentially affected cell population in the different phases of the cell cycle. This novel nanocarrier platform loaded with THQ increased the percentage of cells in the S and pre-G1 phases, while decreasing the population of cells in the G2-M phase. G1-phase arrest of cell cycle progression highlights the apoptotic potential of the novel THQ formulation developed in this study. In accordance with these findings, it has been previously shown that THQ treatment induces apoptosis in human lung adenocarcinoma cells [64]. Yet, the phytosomal delivery of THQ has improved the apoptotic activity of THQ significantly higher than the levels associated with the free drug. The increased apoptotic activity of the THQ formula might be due to enhanced membrane penetration capacity of THQ. This is in agreement with reports in the literature showing that structural modifications of THQ to enhance its cellular internalization resulted in apoptosis and cell cycle arrest in the S-phase colorectal cancer cells [65]. In addition, we found that THQ significantly increased the population of cells in the pre-G phase, which is consistent with other reports in the literature [66]. These findings were confirmed by apoptosis analysis using Annexin V, where the novel THQ formula was found to significantly increase early, late, and total cell death. This is in agreement with previous findings in the literature highlighting the importance of apoptosis in the antiproliferative properties of THQ [67]. This increase in apoptosis associated with the THQ formula could be due to the lipophilic nature of the phytosomes, which can confer improved delivery of THQ to its site of action [68].

THQ phytosomes also significantly increased the mRNA expression of caspase-3, which is consistent with reports in the literature highlighting the potential of THQ to upregulate cellular caspase-3 activity [69]. It is also known that phytosomes augment the targeting and proapoptotic activity of active drugs, including their effects on cleaved caspase-3 content in A543 cells. This was further substantiated by our findings showing a significant increase in the mRNA expression of Bax and a decrease in the mRNA expression of Bcl-2 following treatment of A543 cells with the THQ formula. Treatment with the THQ formula also upregulated the mRNA expression of the tumor suppressor p53. These reciprocal changes in the expression of p53 and the Bcl2 family of proteins are known to induce apoptosis and cell cycle arrest in stressed cells [70,71,72].

Treatment with novel formula was also found to significantly disrupt the MMP, indicating enhanced permeability of the mitochondrial membrane, which is a characteristic feature in apoptosis [42,73]. This induction of p53-dependent apoptosis associated with the THQ formula appears to involve the downregulation of BCL-2 and the upregulation of the apoptotic regulator Bax, which ultimately results in the activation of caspase-3 due to the loss of mitochondrial membrane integrity [74]. In addition, the THQ formula exerted anti-inflammatory effects, as it was found to reduce TNF-α mRNA expression and NF-κB activation. This anti-inflammatory response could be due to the induction of apoptosis by the THQ formula. Apoptotic cells generally trigger anti-inflammatory signaling pathways that eventually result in the inhibition of TNF-α and NF-κB [75,76].

## 5. Conclusions

In the present study, 3^2^ factorial experimental design was implemented to formulate and optimize THQ–PL–SV phytosomes. Prepared formulae exhibited nano-size and high zeta potential. The optimized THQ–PL–SV phytosomes indicated that treatment with THQ formula significantly increased caspase-3, Bax, Bcl-2, and p53 mRNA expression compared to plain formula and THQ. In terms of the inflammatory markers, THQ formula significantly reduced the activity of TNF-α and NF-κB in comparison with the plain formula and THQ only. Overall, the findings from the study proved that a phytosome formulation of THQ could be a promising therapeutic approach for the treatment of lung adenocarcinoma.

## Figures and Tables

**Table 1:** Independent variable levels and response constraints utilized in the 3^2^ factorial design for the optimization of THQ-PL-SV phytosomes.

Independent Variables	Levels		
	(−1)	(0)	(+1)
X1: PL concentration (mg)	79	237	395
X2: SV concentration (mg)	34	102	170
Responses	Desirability constraint		
Y1: Particle size (nm)	Minimize		
Y2: Zeta potential (mV)	Maximize		

Abbreviations: THQ, thymoquinone; PL, Phospholipon^®^ 90H; SV, scorpion venom peptide.

**Table 2:** Combination of independent variables in THQ–PL–SV phytosomes experimental runs, prepared according to 3^2^ factorial design, and their corresponding responses.

Run	X1	X2	Y1	Y2
	PL Concentration	SV Concentration	Vesicle Size * ± SD	Zeta Potential * ± SD
	(mg)	(mg)	(nm)	(mV)
1	79.0	170.0	198.8 ± 9.8	26.8 ± 7.4
2	395.0	34.0	388.0 ± 13.6	2.7 ± 4.1
3	395.0	102.0	412.4 ± 24.1	17.7 ± 8.7
4	395.0	170.0	435.6 ± 19.3	11.8 ± 4.6
5	79.0	34.0	143.7 ± 14.6	4.4 ± 3.9
6	79.0	102.0	176.6 ± 17.9	6.7 ± 3.7
7	237.0	34.0	212.5 ± 13.5	3.8 ± 4.7
8	237.0	170.0	298.8 ± 23.3	9.8 ± 5.2
9	237.0	102.0	274.5 ± 19.9	7.9 ± 6.7
10	79.0	170.0	199.7 ± 21.1	27.1 ± 5.4
11	395.0	34.0	389.7 ± 26.4	2.9 ± 7.3
12	395.0	102.0	410.3 ± 28.7	18.9 ± 5.7
13	395.0	170.0	430.5 ± 23.1	11.8 ± 3.4
14	79.0	34.0	145.8 ± 12.5	4.1 ± 5.3
15	79.0	102.0	174.6 ± 23.8	6.6 ± 4.3
16	237.0	34.0	214.7 ± 18.9	3.8 ± 4.7
17	237.0	170.0	298.4 ± 22.2	9.9 ± 5.1
18	237.0	102.0	276.8 ± 24.3	7.8 ± 3.6

Abbreviations: THQ, thymoquinone; PL, Phospholipon^®^ 90H; SV, scorpion venom peptide; SD, standard deviation. * Results are presented as mean ± SD, *n* = 3.

**Table 3:** Model summary statistics of THQ–PL–SV phytosome responses.

Responses	R^2^ (%)	Adjusted R^2^ (%)	Standard Error of Est.	PRESS	Significant Terms
Y_1_: Vesicle size (nm)	99.3799	99.1216	10.2385	102.10	X_1_, X_2_, $X_{1}^{2}$
Y_2_: Zeta potential (mV)	66.8896	53.0935	5.47816	3.46	X_2_

Abbreviations: THQ, thymoquinone; PL, Phospholipon^®^ 90H; SV, scorpion venom peptide; PRESS, predicted residual error sum of squares.

**Table 4:** Analysis of variance for the vesicle size of THQ–PL–SV phytosomes.

Source	Sum of Squares	Degrees of Freedom	Mean Square	F-Value	*p*-Value
A:X1	169782	1	169782.	1619.63	<0.0001
B:X2	11243.7	1	11243.7	107.26	<0.0001
AA	3485.13	1	3485.13	33.25	0.0001
AB	52.8392	1	52.8392	0.50	0.4925
BB	246.804	1	246.804	2.35	0.1532
blocks	0.0102722	1	0.0102722	0.00	0.9923
Total error	1153.1	11	104.828		
Total (corr.)	185964.	17			

**Table 5:** Analysis of variance for the zeta potential of THQ–PL–SV phytosomes.

Source	Sum of Squares	Degrees of Freedom	Mean Square	F-Value	*p*-Value
A:X1	8.1675	1	8.1675	0.27	0.6122
B:X2	475.021	1	475.021	15.83	0.0022
AA	85.5625	1	85.5625	2.85	0.1194
AB	93.845	1	93.845	3.13	0.1047
BB	4.2025	1	4.2025	0.14	0.7154
blocks	0.0938889	1	0.0938889	0.00	0.9564
Total error	330.113	11	30.0103		
Total (corr.)	997.005	17			

## Data Availability

Not applicable.
